# Clinical management of gastroesophageal junction tumors: past and recent evidences for the role of radiotherapy in the multidisciplinary approach

**DOI:** 10.1186/1748-717X-9-45

**Published:** 2014-02-05

**Authors:** Francesco Cellini, Alessio G Morganti, Francesco M Di Matteo, Gian Carlo Mattiucci, Vincenzo Valentini

**Affiliations:** 1 Radiation Oncology, Policlinico Universitario Campus Bio-Medico, Via Álvaro del Portillo, 200, 00144 Rome, Italy; 2 Radiotherapy Department, Fondazione di Ricerca e Cura “Giovanni Paolo II”, Largo Agostino Gemelli 1, 86100 Campobasso, Italy; 3 Radiation Oncology Department, Policlinico Universitario “A. Gemelli”, Universita` Cattolica del Sacro Cuore, L.go Francesco Vito 1, 00168 Rome, Italy; 4 GI Endoscopy Unit, Policlinico Universitario Campus Bio-Medico University, Via Alvaro del Portillo 200, 00128 Rome, Italy

**Keywords:** Gastroesophageal, Gastric, Esophageal, Review, Radiotherapy, Radiochemotherapy, Chemoradiation, Tumor, Chemotherapy, Management

## Abstract

Gastroesophageal cancers (such as esophageal, gastric and gastroesophageal-junction -GEJ- lesions) are worldwide a leading cause of death being relatively rare but highly aggressive. In the past years, a clear shift in the location of upper gastrointestinal tract tumors has been recorded, both affecting the scientific research and the modern clinical practice. The integration of pre- or peri-operative multimodal approaches, as radiotherapy and chemotherapy (often combined), seems promising to further improve clinical outcome for such presentations. In the past, the definition of GEJ led to controversies and confusion: GEJ tumors have been managed either grouped to gastric or esophageal lesions, following slightly different surgical, radiotherapeutic and systemic approaches. Recently, the American Joint Committee on Cancer (AJCC) changed the staging and classification system of GEJ to harmonize some staging issues for esophageal and gastric cancer. This review discusses the most relevant historical and recent evidences of neoadjuvant treatment involving Radiotherapy for GEJ tumors, and describes the efficacy of such treatment in the frame of multimodal integrated therapies, from the new point of view of the recent classification of such tumors.

## Introduction

Gastroesophageal cancers (such as esophageal, gastric and gastroesophageal junction -GEJ- lesions) are worldwide a leading cause of death, being relatively rare but highly aggressive [1]. Gastric carcinoma was reported as the second cause of cancer-related deaths worldwide [2], and esophageal cancer as the sixth one [3]. In the past years, a clear change of the more frequent locations of upper gastrointestinal (GI) tract tumors has been recorded [1] both affecting the scientific research and the modern clinical practice [4].

Frequency of the stomach’s subsite in which cancer originates, has changed in the recent decades in Western countries [5]. The incidence of cancer of the distal stomach has decreased, while the incidence rate for cancers of the cardia and GEJ has been on a rapid upsurge [6]. Similarly, the most common site of esophageal cancer is the distal one, which often involves the gastroesophageal junction (GEJ) [1]. In developed countries, the incidence of gastric cancer originating from the cardia follows that of the esophageal cancer [7,8], suggesting similar behaviors.

Anatomically, the GEJ is the burden separating the lower esophagus from the proximal part of the stomach, typically in the area where the squamous epithelium of esophagus changes into the columnar epithelium of gastric cardia [9]. Adenocarcinomas of the GEJ represent around 90% of all the GEJ cancers [10], they are generally considered to present lower radiosensitivity than squamous cell carcinomas, but still clear evidences for the specific subset of GEJ tumors are lacking.

Worldwide, the increased incidence of GEJ lesion is homogeneous among western countries like USA, Canada, Sweden, UK and most parts of Europe. The incidence of adenocarcinomas of GEJ, cardia and esophagus increased 4-10% each year since 1976 in USA among men [7,11,12]. Similarly, in Canada an increase of incidence of distal esophageal and proximal gastric adenocarcinomas was recorded from 1964 to 2002 [13]. In contrast, among Eastern Asian countries, the non-gastroesophageal lesions are predominant; as well, squamous cell carcinomas are more frequent than adenocarcinomas [14].

There is a variable incidence of such disease in terms of gender, sex, risk factors and geographical areas of incidence, being Caucasian, male and over 40 years old the most affected subgroup of patients. Predominant risk factors include: gastroesophageal reflux disease (GERD), Barrett’s esophagus and obesity, followed by alcohol abuse and smoking [10,15].

Recent ameliorations of surgical techniques have gained better results over last decades; nevertheless outcomes after resection are still poor, ranging by 20-30% of survival at 5 years, and optimization is needed [16]. The integration of pre- or peri-operative multimodal approaches, such as radiotherapy (RT) and chemotherapy (CT) (also combined), is promising to further improve clinical outcome for such presentations [17].

Purpose of this review is to discuss the most relevant historical and recent evidences on neoadjuvant treatment involving RT for GEJ tumors (in particular Phase III trials), and to describe its efficacy among multimodal integrated therapies, from the point of view of the new classification.

### Classification and staging of gastroesophageal junction tumors

In past, GEJ cancers were alternatively considered as those related with a gastric or esophageal subsite since lying at the anatomical boundary of these two primary sites [18]. Also in clinical trials, GEJ tumors have been managed either grouped to gastric or to esophageal lesions, following slightly different surgical, radiotherapeutic and systemic approaches. Therefore, the definition of GEJ in past led to controversies and confusion [19]. Siewert and colleagues addressed an important attempt of classification for GEJ lesions. In the 80s, a first surgical classification based on clinical preoperative evaluation combining diagnostic and endoscopic features with intra-operative observations was proposed [20,21]. In this classification, three types of GEJ lesions were defined according to the localization of the lesion’s epicenter. The three types of lesion were defined on the range of distance from the GEJ (more than 1 cm above, between 1 cm above and 2 cm below, over 2 cm below that). In the year 2000, the ranges of distance and the details of the definitions were slightly changed [22]. The current Siewert’s classification is summarized in Table 1.

**Table 1:** Siewert’s classification for adenocarcinomas of GEJ

**Siewert type**	**Epicenter of the lesion**
**I**	Within 1 to 5 cm above the anatomic GEJ
**II**	Within 1 cm above and 2 cm below the GEJ (i.e. true carcinoma of the cardia)
**III**	Between 2 to 5 cm below the GEJ, infiltrating GEJ and esophagus from below (subcardial carcinoma)

[GEJ: Gastroesophageal Junction].

In 2006, the same group published the updated long-term results of their series on over 1600 patients [23]. Particularly, they highlighted different survival trends between the three subtypes, with better outcome for types I/II compared to type III. Moreover, they described the nodal spread for each type, particularly useful to guide both surgical dissection and radiotherapy planning (in terms of prophylactic nodal target definition).

The staging system adopted by the American Joint Committee on Cancer (AJCC) is more oriented by pathological findings. Recently, the staging system for GEJ changed, with the aim of harmonizing some staging issues about esophageal and gastric cancer [24]. The International Union Against Cancer (UICC) TNM Classification reports the same changes [25]. In the 7th UICC classification, GEJ tumors (i.e. the Siewert type I-II-III) are grouped as a subsite of esophageal cancer, whose epicenter is: in the distal esophagus, or in the GEJ, or as well within the proximal 5 cm of the stomach (cardia) if extending into the GEJ and distal esophagus. In summary, these three types of Siewert lesions together belong to esophagus according to the new classification. In particular, the Siewert type III lesion is not considered a subsite of gastric cancer any more (unless primary lesion originates within the first 5 cm of stomach but not infiltrating the gastroesophageal junction and esophagus).

Another important change applied by the 7th classification is related to the nodal staging definition. This is now defined by the number of pathologically involved nodes rather than by the location of the involved stations [24] since this issue strongly affects prognosis. For instance, in an experiment on 2920 patients, 5-year survival dropped from 63% in patients without nodal involvement, to 8% in patients with more than 6 pathologically positive nodes [26]. This approach follows the one already in use for gastric cancers [18]. According to some authors, the actual TNM classification does not completely solve the problems for GEJ lesions and remains object of debate, still presenting shortcomings in terms of clarity of interpretation [1].

Some evidences suggest a common behavior between the recently grouped subsites from different points of view. The attention on the similarity of genetic mutation profiles between distal esophagus and GEJ adenocarcinomas is on rise [27].

The lymphatic spread behavior is of major interest in this regard: *Leers et al.* described in a recent paper the risk of nodal involvement for adenocarcinomas from different primary locations [28]. Two groups, one comprising 301 patients with distal esophagus lesions, and the other comprising 208 with GEJ tumors, showed similar rates of nodal spread (47% vs. 41% respectively); the distribution among the nodal station involved and the 5 year survival rates, according to the authors, were also similar. On the other hand, evidences from other series suggest a certain variance of aggressiveness within the GEJ classification, among the three Siewert types. More advanced histologic grades tend to be more frequent in types II and III than those in type I. Moreover, the percentage of involved nodes and the pattern of spread to nodal stations were reported to be slightly different, with a progressive increase of worse presentations from type I to type III [29]. In summary, there are data not only supporting the similarity of GEJ lesions to esophageal (as grouped by TNM classification), but also about the differences among different GEJ lesions on the basis of Siewert’s classification. The nodal spread behavior not only represents a parameter of oncological similarity, and a well known risk factor strongly affecting survival [30-32]: it is also of major importance for radiation oncologist, addressing the treatment volume definition and consequently influencing efficacy and toxicity of RT. Following the surgical experience about anatomical spread types and nodal involvement rates [31,33], many attempts to indicate the optimal coverage of the areas at nodal risk were made for gastroesophageal lesions [34-36]. More recently, important studies from European working group (as the EORTC-ROG) as the one from Matzinger *et al.*[37] and single Institution experience [38], in line with the growing clinical importance of such an issue, have similarly deepened the GEJ lesions, suggesting the optimal target contouring for each Siewert’s type based on risk of involvement. Such considerations support the need to consider both Siewert’s classification and TNM staging tailoring therapies for the different tumor presentations.

### Clinical management

#### Clinical background

Surgery is a major component of treatment for resectable disease. Radical surgery is the most effective treatment modality, with the aim of the en-bloc removal of primary tumor and lymphatic nodes, obtaining microscopically negative resection margins (R0) [19]. In large historical surgical series, survival was strongly affected by the accomplishment of R0 resection: among 1602 patients, Siewert reported a 5-year survival rate of 43.2% vs. 11.1% for R0 and positive margins, respectively (p < 0.0001) [23].

One of the major developments of surgery was the clear reduction in surgical morbidity and mortality as a result of improvements in staging techniques, patient selection and support therapies. Nowadays, surgery alone is mostly adopted for the treatment of early localized presentations [1].

The efficacy of neoadjuvant integrated treatments has been widely investigated over last the decades to overcome the poor outcome of more advanced presentations.

A recently published meta-analysis from 17 randomized trials including neoadjuvant RT, chemotherapy (CT) and surgery for esophageal carcinoma, strongly supported the adoption of combined-modality treatments [39]. Selected trials included both neoadjuvant CT and RT: the latter was administered as concomitant radiochemotherapy (RTCT) or following CT (in older studies). Results addressed a clear benefit from both RTCT (p < 0.0001) and CT (p = 0.005) over surgery alone, and a slightly non-significant trend favoring RTCT respect to CT (p = 0.07). The absolute benefit was defined as an improvement of 2-year survival of 8.7% and 5.1%with RTCT and CT, respectively.

International Guidelines still do not completely agree on the standard treatment approach but basically suggest similar treatment options for adenocarcinoma of the esophagus (that nowadays the GEJ tumors belong to). The National Comprehensive Cancer Network (NCCN) recommends surgery alone for the very early presentations (i.e. Tis, T1a,T1bN0), and preoperative RTCT for all the others [1]. The National Cancer Institute (NCI) in its Physician Data Query (PDQ) suggests preoperative RTCT for presentation from Stage I to IV (non-metastatic) [40]. The European Society for Medical Oncology (ESMO) indicates: surgery for early presentations; perioperative chemotherapy for localized disease or, alternatively, preoperative RCTC. Preoperative RTCT and perioperative CT are recommended for locally advanced disease [41].

#### Radiotherapy for gastroesphageal junction tumors:historical evidences

Over the past 3 decades, the efficacy of RT for GEJ tumors was progressively clarified.

**Arnott et al. in 1998** published a meta-analysis based on 5 available randomized trials of RT (associated to CT or not) versus Surgery [42]. They reported a non-statistically significant absolute survival benefit of 3% at 2 years and 4% at 5 years (HR: 0.89; CI 0.78-1.01; p = 0.062). It has to be stressed that the global accrual period of the reported trials ranged from 1973 to 1988, suggesting the use of older technology of treatment planning and dose delivery: some of the trials even used a Cobalt treatment unit. Moreover, the RT dose ranged from 20 to 40 Gy among the studies (lower than the most commonly used nowadays). The main characteristics of this and other meta-analyses mentioned in this review are summarized in Table 2.

**Table 2:** Meta-analyses on preoperative treatment (RT; RTCT; CT) versus surgery

	**Primary tumor site**	**Range of recruitment periods**	**N° trial**	**N° trial**	**N° total pts**	**N° pts RT±CT arms**	**N° pts CT arms**	**N° pts surgery arms**	**Dose range (Gy)**	**Hystology**	**Hazard ratio**	**SVV benefit**	**SVV benefit**
				**Including RT**							**(95% CI ; p)**	**2 yy**	**5 yy**
Arnott [42]	Esophagus	1973-1988	5	5	1147	573	-	574	20-40	SCC-86%	0.89	4%	3%
						(520 RT alone + 53 RTCT)				ADC-14%	(CI 0.78-1.01; p=0.06)		
Fiorica [47]	Esophagus	1983-1995	6	6	764	385	-	379	20-45	SCC-76%	0.53	NS	NS
										ADC-24%	(CI 0.31-0.92; p=0.03)		
Sjoquist [39]	Esophagus	1982-2008	24	14	4188	1079	1046	2063	20-50.4	SCC-48.9%	RTCT: 0.78	RTCT: 8.7%	NR
											(CI 0.70-0.88; p<0.0001)		
										ADC-35.5%	CT: 0.87	CT: 5.1%	
											(CI 0.79-0.96; p=0.005)		
											RTCT vs CT: 0.88		
											(CI 0.76-1.01; p=0.07)		
Ronellenfitsch [16]	Esophagus + Stomach + GEJ	1987-2004	14	4	2422	198	1024	1200	35-50.4	SCC-0%	CT (±RT):0.81	NS	CT (±RT):9%
											(CI 0.73-0.89; p<0.0001)		
										ADC-100%	RTCT: 0.70		
											(CI 0.50-0.99; p=0.38)		
											CT: 0.83		
											(CI 0.75-0.91; p=0.38)		

[Pts: patients; Gy: Gray; pCR: pathological complete response; RT: Radiotherapy; CT: Chemotherapy; RTCT: radiochemotherapy; yy: years ; CI: Confidence Interval; SVV: survival; SCC: Squamous Cellular Carcinoma; ADC: Adenocarcinoma; GEJ: Gastroesophageal Junction; NS: Not Specified; NR: Not Reported].

**Walsh *et al.*** **in 1996** published a landmark randomized study on esophageal tumors addressing the role of RT for GEJ adenocarcinomas. Cardiac tumors (at least considerable as Siewert II-III), or middle and lower third esophageal cancers (the latter is considerable at least in part as a Siewert I: details were not reported in detail by authors) [43]. This trial was not included in the Arnott’s meta-analysis [42]. Between 1990 and 1995, 113 patients affected by adenocarcinoma were enrolled and randomized to either RTCT followed by surgery or surgery alone. The rate of patients affected by lesions of the middle third of the esophagus was 14.1%, the remaining 85.9% are at least considerable GEJ in the modern classification. Patients in the experimental arm received RTCT (with 5-Fu and CDDP) up to a dose of 40 Gy (with 2.7 Gy per fraction in 15 fractions over 3 weeks). Of the 52 patients who underwent surgery in the RTCT arm, 13 (25%) presented pathological complete response (pCR). Median survival was 16 and 11 months (mth) for preoperative RTCT and surgery-only arm (p = 0.01), respectively. The 1-, 2- and 3-year survival rates were 52%, 37%, and 32% in the RTCT arm, and 44%, 26% and 6% in the surgery-only one, respectively (p = 0.01).

Between 1998 and 1999, two randomized trials testing the superiority of RTCT over RT alone in the preoperative setting of esophageal tumors were published [44,45]. These studies are not really representative of GEJ adenocarcinomas due to their eligibility criteria. **Smith *et al.*** enrolled approximately 50% of the patients with the primary site of tumor in the lower third of esophagus, but all patients presented SCC lesion; **Cooper *et al.*** enrolled all patients with primary site in the thoracic esophagus. Anyway, these trials encouraged the approach of following experiences for esophageal cancer, towards RTCT in the preoperative setting [17].

In **2001, Urba *et al.*** published a randomized trial on preoperative RTCT versus surgery only [46] on patients presenting tumor of the esophagus or GEJ. It is difficult to precisely define the rate of GEJ tumor in patients enrolled in this trial, according to the modern definition. Ninety-two percent of patients in both arms had a middle or lower third lesion, with a rate of upper third lesions lower than 10%. Adenocarcinoma accounted for 75% (only 24% of squamous cell carcinoma). One hundred patients were enrolled from 1989 to 1994: 50 patients received RTCT with a total dose of 45 Gy (1.5 Gy per fraction, two fraction per day, over 21 days), concurrently with 5-FU, CDDP and vinblastine. In the surgery-only arm 90% (45/50) had a gross total resection; and in the RTCT arm 95.7% (45/47 who underwent surgery) had a gross total resection. In the experimental arm, 28% had a pCR (14/50, according to intention to treat). Median survival and 3-year survival for surgery and RTCT arms were 17.6 vs. 16.9 mth, and 16% vs. 30% (p = 0.15), respectively. It has to be noted that this study was statistically powered to detect a relatively large increase in median survival, and that the dose fractionation and CT association differed from the more common standards. Interestingly, when considering the survival by pathological response, a clear significant benefit was found: the reported median survival and 3-year survival rate for the pCR group versus the non-pCR were 49.7 vs. 12 mth, and 64% vs. 19%, respectively, highlighting the importance of preoperative effective treatment for responder patients.

**Fiorica *et al.*** **in 2004** published a meta-analysis including 6 trials of RTCT versus surgery alone [47]. This study included the two papers by Walsh *et al.* and Urba *et al*. mentioned above.

Authors not only showed a significant survival benefit of neoadjuvant RTCT over surgery alone (HR: 0.53; CI: 0.31-0.92; p = 0.03), but also highlighted a significant risk for postoperative mortality. Two considerations should be addressed. First, the reported survival benefit was still not strong in itself. Survival evidence of benefit was lost for the exclusion from the statistical evaluation of either the study from Walsh, or the one from Urba. Therefore, the need for further evidence was clear. Second, the toxicity related to neoadjuvant RTCT was mainly due to the contribution of a trial by Bosset *et al.*, using non-conventional RT doses and fractionations, also questioned by its Authors, and no longer used in the following experiences [17] (see also Table 2).

#### Recent randomized trials of radio(chemo)therapy plus surgery versus surgery alone

**Burmeister *et al. *** **in 2005**[48] failed to report a significant benefit adding preoperative RTCT to surgery alone. Between 1994 and 2000, they randomized 256 patients. Lesions were cited in the proximal or middle esophagus for 23% in the RTCT arm and for 19% in the surgery alone one. Distal esophageal lesions (more representative for GEJ) accounted for 77% in the RTCT arm and for 81% in the surgery alone one. Primary lesions originating in cardia with predominant esophageal invasion (similar to Siewert III) were included. Adenocarcinomas were approximately 60% in both arms. RTCT delivered 35 Gy (in 15 fraction of 2.4 Gy over 3 weeks) with concurrent 5-FU and CDDP. RTCT was well tolerated: esophagitis was the highest Grade 3-4 toxicity, reported in 16% of patients. There was no significant benefit for progression free and overall survival in the RTCT arm respect to the control one, even though a small trend favoring RTCT was reported. It is interesting to highlight that in the experimental arm, a significantly higher R0 resection rate (80% vs. 59%; p = 0.0002), and lower rate of pathologically positive lymph-nodes (43% vs. 67%; p = 0.003) were reported. Patients in the RTCT arm reported 16% rate of pCR. It should be highlighted that the RT dose was lower than that in the other modern randomized trials that reported an outcome improvement.

**In 2008, Tepper *et al.*** published a trial on patients with esophageal or GEJ tumor (both squamous and adenocarcinomas) [49]. Lesions of the thoracic esophagus (below 20 cm), and/or GEJ with less than 2 cm distal spread into the cardia (the latter corresponds to the actual Siewert II) were included. The rate of lesions in the thoracic esophagus was not specified, but 75% of the 56 enrolled patients had adenocarcinoma (orienting for a major presence of GEJ lesions). Between 1997 and 2000, 30 patients in the experimental arm received RTCT with a total dose of 50.4 Gy (45 Gy in extended field + 5.4 Gy as boost; conventional fractions over 5.5 weeks) plus concomitant 5-FU and CDDP. Grade 3-4 esophagitis was the highest non-hematological toxicity, reported in 26% for RTCT. Pathological data were available only for 25/30 patients in the RTCT arm: pCR was reported for 40% (10/25). At a median follow-up (fup) of 6 years, according to intent-to-treat analysis there was a significant survival benefit for RTCT over surgery alone. Median survival was 4.48 vs. 1.79 years and 5-year survival was 39% vs. 16% for RTCT and surgery alone, respectively (p = 0.002). The study was prematurely closed due to poor accrual (respect to the 475 planned), and suffered for this statistical bias.

**Van Hagen ***et al. ***in 2012** published the “Chemoradiotherapy for esophageal cancer followed by surgery study” (CROSS) trial, advocated as potential gold standard for esophageal cancer [50]. Between 2004 and 2008, 366 patients affected with esophageal or GEJ carcinoma were enrolled. Pure GEJ lesions were reported in around 25% of patients. Over 80% of the patients in both the arms had primary lesion cited in the distal third lesion or at the GEJ, and adenocarcinomas accounted for 75%. RTCT consisted of a dose of 41.2 Gy (conventional fractionation, over 5 weeks) plus weekly Carboplatin and Paclitaxel. Tolerance to RTCT was good: 91% of patients in experimental arm received the full treatment regimen. In the RTCT group, 94% of patients underwent surgery versus 99% in the control arm (p = 0.01); seven patients in RTCT group showed disease progression during treatment, compared to one in the surgery-only group. An R0 resection was obtained for 148/161 patients (92%) versus 111/161 (69%) in the RTCT and control group respectively (p < 0.001). At a median fup of 45.4 mth, the 3- and 5-years overall survival rates were 58% vs. 44% and 47% vs. 24% for combined treatment and surgery alone arms, respectively (p = 0.003).

Two of the most interesting pathological findings should be highlighted. The first is the 29% rate of pCR (47/161) in the RTCT group, higher but in line with the previously published evidences for GEJ tumors. Second: the significantly lower presence of pathological nodal involvement in the RTCT compared to the surgery only arm: 31% vs. 75% respectively (p < 0.001).

In 2014, a specific analysis of the relation between the site of recurrence and radiation field in CROSS I and II trials was published [51]. **Oppedijk et al.** showed that RT significantly improved over surgery alone both locoregional recurrence (14% vs. 34%; p < 0.001) and peritoneal carcinomatosis (4% vs. 14%; p < 0.001), also presenting a small but significant benefit on hematogenous dissemination rates (29% vs. 35%; p = 0.025). Recurrences within the RT treatment volume occurred in only 5% (11/213) evaluable patients.

The main randomized trials of RTCT plus Surgery versus Surgery alone are summarized in Table 3.

**Table 3:** Phase III randomized trials comparing radiochemotherapy plus surgery versus surgery alone

	**N° Pts**	**Accrual**	**Rate adeno**	**Tumor site**	**Dose/Fx (Gy)**	**Concurrent CT**	**% pCR**	**3 yy OS %**	**5 yy OS %**	**Median SVV (mth)**	**Median fup (mth)**
							**(N° pts RTCT arm)**	**[RTCT+ surg vs. surg alone]**	**[RTCT+ surg vs surg alone]**	**[RTCT+ surg vs surg alone]**	
Walsh [43]	113	1990-1995	100%	Middle+ Lower Esophagus + Cardias	40/2.7	CDDP + 5Fu	25% (13/52)	32 vs. 6	-	16 vs 11	10 (0.1-59)
								(p=0.01)			
Urba [46]	100	1989-1994	75%	Proximal+ Middle + Lower Esophagus + GEJ	45/1.5 (twice daily)	CDDP+ 5Fu+ Vimblastine	28% (14/50)	30 vs. 16	-	16.9 vs 17.6	98.4 (72-118.8)
								(p=0.15)			
Burmeister [48]	256	1994-2000	62%	Proximal +Middle+ Lower Esophagus	35/2.4	CDDP + 5Fu	16% (16/103)	42 vs. 36	21 vs. 19	22.2 vs. 19.3	65 (0.4-120)
								(p=0.57)			
Tepper [49]	56	1997-2000	75%	Toracic Esophagus (below 20 cm)+ GEJ <2cm distal spread in cardia	50.4/1.8	CDDP + 5Fu	40% (10/25)	-	39 vs. 16 (p=0.002)	53.8 vs. 21.5	72 (NR)
Van Hagen [50]	366	2004-2008	75%	Proximal +Middle+ Lower Esophagus + GEJ	41.2/1.8	Carboplatin + Paclitaxel	29% (47/161)	58 vs. 44	47 vs. 34	49.4 vs. 24	45.4 (25.5-80.9)
								(p=0.003)			

[Pts: patients; Gy: Gray; pCR: pathological complete response; RTCT: radiochemotherapy; yy: years; OS: overall survival; Surg: surgery; SVV: survival; mth: months; Fup: follow-up; CDDP: Cisplatin; 5Fu: 5fluoruracil;].

#### Randomized trials of radio(chemo)therapy versus chemotherapy

Two randomized trials recently compared RTCT versus CT in the preoperative setting.

**Burmeister** ***et*** *al.* randomized in a phase II trial, between 2000 and 2006, 75 patients presenting resectable adenocarcinoma of the esophagus and GEJ. The planned accrual was 100 patients, but was reduced due to poor accrual in the RTCT arm for technical reasons. Treatment consisted of either CT with 5-FU and CDDP, or RTCT delivering a dose of 35 Gy (in 15 fractions of 2.4 Gy over 3 weeks) concomitantly to the same drugs at reduced doses [52]. Similarly to their previous trial comparing RTCT to surgery alone [48], RT dose was lower than in other reported trials: again RTCT failed to significantly improve outcomes. Nevertheless, in the RTCT group a positive trend was reported for progression free survival (14mth-CT vs. 26 mth-RTCT; p = 0.37) and overall survival (29mth-CT vs. 32mth-RTCT; p = 0.83). It is interesting that the RTCT approach gained significantly higher pathological response rates (RTCT 31% versus CT 8%; p = 0.01) and R1 resection rates (RTCT 0% versus CT 11%; p = 0.04). Toxicity profiles were similar in the 2 arms. The statistical power of this study should be interpreted with caution, especially in regard to progression free survival. Authors concluded that the most important evidence was about the not increased toxicity and the superior pathological response in the combined arm.

**Stahl** ***et al.*** published in 2009 a phase III trial on 126 patients (enrolled between 2000-2005) affected by adenocarcinoma of the lower esophagus and cardia (Siewert I-III) [53]. Treatment consisted of either 15 weeks of CT (with CDDP, 5-Fu, Leucovorin) or 12 weeks of the same CT followed by 3 weeks of RTCT (30 Gy with conventional fractionation plus CDDP and Etoposide). In the RTCT arm, a significant improvement of the pCR rate (RTCT: 15.6% vs. CT: 2.0%; p = 0.03) was reported. Also the tumor-free lymph-nodes rate was significantly higher in the RTCT group (RTCT: 64.4% vs. CT: 36.7%; p = 0.01). Preoperative RT showed a positive non-significant trend in the 3-year survival rates (RTCT: 27.7% vs. CT: 47.4%; p = 0.07). It is important to stress as the non significance of such results should be carefully interpreted for some reasons: the study was statistically designed to detect a 10% improvement in the 3 year survival for the RTCT arm, expecting a much larger accrual in each arm; almost a 20% of improvement was actually reported, but the total number of needed patients after the planned first stage of the protocol required 163 additional patients per arm, then the study was closed without obtaining the required statistical power. Moreover, the impact of the pathological findings reflected the subgroup survival analysis: the pathologically node-negative patients had a significantly higher 3-year survival (64.2% vs. 38.8%; p < 0.001). Patients achieving pCR were all alive at the median follow-up time of 4.1 years, independently by administered treatment.

About the tolerance of preoperative RTCT, a non-significantly increased postoperative mortality in the RTCT arm (10.2% vs. 3.8%; p = 0.26) was reported: this data is not completely in line with the others experiences in literature (in particular the available meta-analyses suggest a risk of postoperative morbidity slightly decreasing in the more recent trials), and the 12 weeks of CT induction before RTCT could have played a role.

A final consideration on the study by Stahl should be addressed about the absolute pCR rate reported in the RTCT arm. Even though the pCR rate in RTCT is significantly higher than that in the CT arm, it is considerably lower than that in the previously mentioned series. It is arguable (as suggested by the authors) that the low RT dose had an influence on that aspect. It could also be hypothesized that the choice of the RT dose was influenced by attempt to keep the overall duration of the RTCT treatment similar to the CT one (i.e. 15 weeks).

We have already highlighted, among the previously cited clinical experiences, that inducing a pCR after neoadjuvant treatment is crucial for the prognosis. This is a known issue, highlighted from historical series of specific experiences on both esophageal and gastric tumors [54-56]. That issue was also recently confirmed in one of the largest single center retrospective series, published by Fields *et al*. [57]. They reviewed cases from 714 patients treated between 1985 and 2009 at the Memorial Sloan-Kettering Cancer Center: all of them were treated with preoperative therapy (i.e. CT with or without RT) for localized gastric or GEJ adenocacinoma. On 714 treated patients, 609 had an R0 resection after preoperative treatment. The vast majority of R0 patients had GEJ lesion (359/609 -60% of pure GEJ only; 439/609-72% of pure GEJ + proximal gastric lesions). A whole rate of 8.4% (60/609) of pCR was reported. These patients showed significantly lower rate of 5-year recurrence compared to non-pCR patients (27% versus 51%; p = 0.01). It is arguable that the low absolute rate of pCR is due to the long time frame of evaluation, meaning older RT treatments and drugs administered.

**Ronellenfitsch *et al.*** published in 2013 a meta-analysis based on aggregate and individual patient data collecting randomized phase III trials comparing surgery alone versus surgery preceded by neoadjuvant treatment at least including CT (either alone or combined with RT). It summarizes some issues debated in this review. Authors collected 14 phase III trials on adenocarcinoma, of esophagus, GEJ or stomach: time to death from randomization, on an intention-to-treat base was the primary outcome [16]. They highlighted that administration of CT (globally evaluated as preoperative CT alone + RTCT) gained significantly longer survival respect to surgery alone, with an absolute benefit of 9% at 5-year survival (HR 0.81, CI 0.73-0.89; p < 0.0001). Moreover, a longer disease free survival, higher down staging rate and R0 resection probability were reported. Preoperative therapy was not associated to higher postoperative complication likelihood. When separately analyzed by anatomical subsites, the reported results were largest for the GEJ presentations, followed by esophageal and gastric tumors. In direct subgroup analysis, RTCT showed a larger (but not significant) effect then CT in producing the survival benefit (HR: 0.70, CI 0.50-0.99 for RTCT vs. 0.83, CI 0.75-0.91 for CT; p = 0.38). Authors summarize that the available evidences suggest a benefit from adding RT to CT for adenocarcinoma of GEJ and esophagus.

It is also relevant to note that due to the timing of the meta-analysis, the CROSS trial from Van Hagen [50] was not included, and it is arguable that it could have enhanced the statistical power.

Sign of the interest and relevance of the issue of preoperative RTCT for adenocarcinoma of the pure GEJ is provided by the growing number of new clinical experiences focused on it, involving the new RT technologies, and providing positive results: Platz et al. reported in 2013 as intensity-modulated-RT (IMRT) up to 50 Gy plus CT, produced 100% of R0 resection rate and 38% of pCR in a group of 16 patients [58]. At a median follow-up of 15.3 mth (9.8-20), 3/16 patients had recurrence (1 anastomotic, 2 distant nodal). Moreover, some important PhaseII/III studies focused on preoperative RTCT on GEJ tumors are currently registered and on-going, as the TOPGEAR trial from the collaboration of EORTC -TROG (Identifier: NCT01924819), randomly evaluating perioperative CT associated or not to preoperative RTCT. At least 2 other studies are focused on preoperative RTCT in the specific population of GEJ lesion only lesions: one from a Chinese group in Hebei (Identifier: NCT01962246) evaluating RTCT versus CT alone for Siewert types II-III; the other from a Polish one in Lublin (Identifier: NCT01523015) comparing RTCT (for type I-II) or CT alone (for type III) plus surgery versus surgery alone. In line with the growing trend of research on the new molecular targeted agents for GEJ tumors, studies are on-going specifically recruiting patients with GEJ lesions. Many biologically targeted agents are under evaluation, but again the research scenario is still often recruiting GEJ tumors together with esophageal and gastric ones. Evidences seem more than promising, particularly oriented to drugs targeting EGFR, VEGF and HER, this last one seems one of the most promising agents based on the still few evidences from direct evaluation of these drugs in concomitant combination with RT [4]. Still more robust evidences are needed to clearly define the optimal strategy, moreover we must be aware of the difficulties in defining response profiles: attempts in this direction are addressed to individuate genetic signatures profiling the GEJ tumors [59,60], their response to therapies [61] or specific genes and molecules potential candidates for new drug development [62]. Finally, also the presence of specific histological patterns as signet ring cells or mucinous histology could be predictive of worse prognosis and better response to RTCT [63,64] and should be included in the treatment decision process or new trial setting, but evidences in this regard are still not conclusive [65].

## Conclusions

Looking at the main evidences reported in literature about the role of RT in the multimodal management of GEJ adenocarcinomas, some problems are still evident in term of clarity of the classification, patient selections in the trial and evaluation of results. Nevertheless, we can summarize that the integration of preoperative RT before surgery is highly effective over surgery alone, providing significantly improved results in terms of local control and survival, with acceptable toxicity rates. In this setting, RT should be administered at least to a total dose of 40-45 Gy (with conventional fractionation), and associated to concomitant chemosensibilization. A non-significant positive trend gaining better results over preoperative CT alone is also reported in literature. To define the best treatment option, a new randomized trial, with a particular attention to accrual criteria, taking into account both TNM and Siewert’s classification is needed. To tailor the future treatment approaches, it is crucial to consider the molecular profile of the disease and include the more promising new molecular targeted therapies, as well as considering the new option of RT delivering coming from new technologies.

## Abbreviations

5-Fu: 5-Fluoruracil; CT: Chemotherapy; CDDP: Cisplatin; fup: Follow-up; GEJ: Gastroesophageal junction; GI: Gastrointestinal; GERD: Gastroesophageal reflux disease; mth: Month; OS: Overall survival; pCR: pathological complete response; RT: Radiotherapy; RTCT: Radiochemotherapy.

## Competing interests

The authors declare that they have no competing interests.

## Authors’ contribution

FC: designed the paper setting, selected evidences, extracted data and drafted paper; AGM: supervised and edited the paper drafting, paragraph setting, and evidences selection;FMD: supervised the classification section; GM: supervised and edited the trial section; VV: supervised and edited the paper setting and evidence selection; All authors read and approved the final manuscript.
